# Little Praise for "In praise of forgiveness"

**DOI:** 10.5964/ejop.5571

**Published:** 2021-11-30

**Authors:** Catalina Woldarsky Meneses

**Affiliations:** 1 Psychology and Counselling Department, Webster University, Geneva, Switzerland; Department of Psychology and Counselling, Webster University Geneva, Geneva, Switzerland

**Keywords:** forgiveness, psychoanalysis, intimate relationships

## Abstract

This is a review of Massimo Recalcati's book "In Praise of Forgiveness" (Polity Press, 2020). It outlines Recalcati’s position on the process of forgiveness and offers a critique of various aspects of this book.

*In Praise of Forgiveness*, is a book written by Massimo Recalcati (2020) that exalts forgiveness as the answer to the pain stemming from betrayals within the context of romantic relationships. The central argument of this book is that forgiveness is a maturational experience requiring the bruised Ego to go beyond itself and see the Other as who he or she truly is rather than some idealized version of him or herself. The thesis, however, is overshadowed in the first part of the book by the author’s critique of modern love, in particular the confusion between love and lust, and the endless quench for novelty. Recalcati’s gender-normative descriptions of love and relationships are highly problematic on many levels, and immediately distort the focus of the book. Written from a psychoanalytic perspective and heavily influenced by the Christian position of “turning the other cheek” (biblical references are included), Recalcati insists that forgiveness offers the injured partner an opportunity for growth that allows a for a more sophisticated and mature appreciation of love and life. According to Recalcati (2020),

“The work of forgiveness is not fed by narcissistic infatuation with one’s own ideal image.. it does not confront the subject with the ideal image of the other but with its most real real of the Other.. bringing us closer to the mystery of the total recalcitrance of the Other, of its being irreducibly foreign, *heteros.”* (p. 90).

The author relies on classical Freudian concepts to explain how forgiveness leads to a profound personal change in the injured partner’s perception of the other but does not actually offer a clear definition of what forgiveness is, how the resolution process unfolds, or how it can be facilitated in the context of therapy. In addition, Recalcati does not include research, nor does he present existing works by other psychoanalytic writers on the subject of forgiveness. It is an unfortunate oversight that Recalcati does not make reference to Siassi’s (2013) book in which forgiveness is also framed as a maturational experience, one that is considered to be so deep that it goes beyond religious imperatives. It would have been interesting for Recalcati to reflect and comment on the arguments presented by Siassi (2013).

*In Praise of Forgiveness* idealizes forgiveness as the best moral choice and fails to discuss or even acknowledge the dangers of promoting forgiveness. There are situations in which forgiveness may be contra-indicated (e.g., in situations of ongoing abuse). Forgiveness researchers, Woldarsky Meneses and Greenberg (2019) assume a “non-authoritarian” perspective on forgiveness and highlight that choosing to not forgive is an empowering choice that should be seen as a legitimate action in itself and depending on the situation it may be the most emotionally authentic course of action.

“Learning not to forgive, after a life in which forgiveness has been compulsive, imposed or unconsidered, is an impressive achievement… Choosing *not to forgive* however can differ from holding onto a grudge in that it can have involved profound self-examination, just like forgiveness-only with a different conclusion.” (p. 22).

Finally, a major point of contention of Recalcati’s book that cannot be overlooked is his sexist and simplistic view of human functioning. For example, the author begins by establishing that there is a core difference between a woman’s way of experiencing romantic love (erotomania) and that of a man (fetishism). He then offers a personal anecdote to support his position and insists that it is this fundamental difference that leads to so many problems in romantic relationships. This reductionistic, hetero-normative view of men and women, as well as the simplistic understanding of intimate relationships is the first challenging aspect of the book that the reader encounters. This position becomes more apparent as he discusses betrayals, and then highly troublesome in the final section of the book, focused on the work of forgiveness where he includes numerous sub-sections that reinforce his sexist position. In the section on *Why men find it more difficult to forgive*, Recalcati (2020) argues that “phallic pride usually opposes the work of forgiveness” (p. 93) and outlines how men’s fear of castration surpasses that of women “for whom castration belongs, as it were to the structure of her being” (p. 93), and therefore they have more difficulty accessing vulnerability. The consequence, he suggests, is that men will seek justice or express violence. Recalcati’s position on gender-based violence is revolting, as captured in the following reflection that he puts forth after briefly describing a woman he saw in therapy who returned to an abusive relationship in what he suggested to be feminine masochism:

“Why do women choose the worst for their love lives? Why do they throw themselves into the arms of men who treat them only as objects? For many women, the difficulty of subjectivizing their otherness – the difficulty of being women – is falsely resolved by throwing themselves into the arms of men who offer the illusion of being compasses capable of guiding their path before transforming themselves into predatory tyrants.” (p. 97)

In sum, *In Praise of Forgiveness* is an example of misogynist literature that can be openly published in academia because it is includes references to Freud, Lacan and other men whose theories reinforce the patriarchy. The transformative process of forgiveness is reduced to commentary about the shift in the views between Self and Other following a betrayal, without any support from the literature. This book is challenging to read for those who are not well-versed in psychoanalytic theory and its extensive lexicon, as well as for anyone with feminist views.
